# Molecular Characterization of a Novel Shell Matrix Protein With PDZ Domain From *Mytilus coruscus*

**DOI:** 10.3389/fphys.2020.543758

**Published:** 2020-10-02

**Authors:** Qi Sun, Yuting Jiang, Xiaojun Yan, Meihua Fan, Xiaolin Zhang, Huanzhi Xu, Zhi Liao

**Affiliations:** Laboratory of Marine Biology Protein Engineering, Marine Science and Technical College, Zhejiang Ocean University, Zhoushan, China

**Keywords:** *Mytilus coruscus*, shell matrix proteins, PDZ-domain-containing protein, recombinant expression, biomineralization

## Abstract

Mollusk shells are products of biomineralization and possess excellent mechanical properties, and shell matrix proteins (SMPs) have important functions in shell formation. A novel SMP with a PDZ domain (PDZ-domain-containing-protein-1, PDCP-1) was identified from the shell matrices of *Mytilus coruscus.* In this study, the gene expression, function, and location of PDCP-1 were analyzed. PDCP-1 was characterized as an ∼70 kDa protein with a PDZ (postsynaptic density/discs large/zonula occludes) domain and a ZM (ZASP-like motif) domain. The PDCP-1 gene has a high expression level and specific location in the foot, mantle and adductor muscle. Recombinantly expressed PDCP-1 (rPDCP-1) altered the morphology of calcite crystals, the polymorph of calcite crystals, binding with both calcite and aragonite crystals, and inhibition of the crystallization rate of calcite crystals. In addition, anti-rPDCP-1 antibody was prepared, and immunohistochemistry and immunofluorescence analyses revealed the specific location of PDCP-1 in the mantle, the adductor muscle, and the aragonite (nacre and myostracum) layer of the shell, suggesting multiple functions of PDCP-1 in biomineralization, muscle-shell attachment, and muscle attraction. Furthermore, pull-down analysis revealed 19 protein partners of PDCP-1 from the shell matrices, which accordingly provided a possible interaction network of PDCP-1 in the shell. These results expand the understanding of the functions of PDZ-domain-containing proteins (PDCPs) in biomineralization and the supramolecular chemistry that contributes to shell formation.

## Introduction

Mollusk shell, which has significant value in the fields of bioengineering and bionics, consists of an outer organic periostracum and inner mineral layers (Barthelat, 2010; Li et al., 2017). The periostracum constitutes the “skin” covering on the whole shell outside (de Paula and Silveira, 2009; Wählisch et al., 2014), and the shell mineral layer has excellent mechanical properties and is mainly composed of calcium carbonate crystals (Arivalagan et al., 2017). In addition, shell contains a low abundance (<5%) of organic matrices consisting principally of shell matrix proteins (SMPs), which play important roles in the formation and mechanical properties of the shell, although the underlying mechanisms have not yet been revealed in detail (Kocot et al., 2016). During shell formation, calcium carbonate crystals are deposited, form nano-structures with different morphologies and polymorphs under the control of SMPs and assemble into a complete shell (Meenakshi et al., 1975; Kocot et al., 2016). Structural and functional studies of SMPs will help to understand the process of shell formation, as well as the molecular mechanism of the excellent mechanical properties of shells.

*Mytilus coruscus* is a mussel with important economic value in the East China Sea. Its shell is composed of three layers, namely, nacre, myostracum, and fibrous prism, and it contains more than 60 SMPs previously identified by transcriptome-proteome strategies (Liao et al., 2015). Within the shell proteome, a novel SMP (PDZ-domain-containing-protein-1, PDCP-1, GenBank No. AKS48171.1) was identified with a PDZ domain from the myostracum layer (Liao et al., 2015). The myostracum layer is exposed on the shell inner surface where the adductor muscle is attached, forming the adductor muscle scar (AMS) of each shell valve (Lee et al., 2011; Liao et al., 2015). The myostracum layer plays an important role in muscle-shell attachment, controlling the closure of shells. However, studies on the organic matrix and the structural roles of the myostracum layer are few in number (Lee et al., 2011). To date, only a few studies have revealed the molecular composition of SMP from the myostracum of *Mytilus*, including *M. coruscus* (Liao et al., 2015) and *M. edulis* (Gao et al., 2015). Interestingly, most identified myostracum-specific SMPs have a potential actin-binding domain, such as vWA (Song et al., 2012), PDZ (Banerjee and Wedegaertner, 2004), calponin (Leinweber et al., 1999), calponin-homology (Magill et al., 2016), and filamin (Miyake et al., 2019), indicating a possible interaction network between SMPs and actin in the myostracum layer. Recently, a whirlin-like protein (WLP), a myostracum-specific SMP with a single PDZ domain, was identified in the shell of *M. coruscus*, and it exhibited biomineralization-related functions and actin-binding ability (Jiang et al., 2020). These results suggested that SMPs of myostracum could play a role in both myostracum formation and interaction of this layer with adductor muscle, a tissue with abundant actin.

PDCP-1 contains a PDZ domain and a ZM domain and exhibits a similar sequence to that of PDZ and LIM domain proteins (PDLIMs) from other species with sequence identity < 40%. PDLIMs have been identified from invertebrates to vertebrates with multiple functions, and all share a PDZ domain (Krcmery et al., 2010). In general, PDLIMs are structural proteins with wide distribution in organisms and play important roles in cytoskeletal organization, organ development, and neural signaling (Cui et al., 2019). The PDZ domain, with a highly conserved sequence of approximately 80–120 residues, is one of the most common modules involved in protein-protein binding (Manjunath et al., 2018). PDZ domains are specialized for binding to the C-terminal peptide motifs of other proteins (Harris and Lim, 2001) and recognize a wide variety of proteins that form multiple protein complexes with multiple ligands (Te and Bagowski, 2007). In addition, the ZM domain is a short motif (26 amino acids) first found in the alpha-actinin-binding protein ZASP (Z-band alternatively spliced PDZ-motif protein), and it is frequently found in association with PDZ domains in a number of cytoskeletal and muscle proteins (Faulkner et al., 1999; Klaavuniemi et al., 2004). PDZ-domain-containing proteins (PDCPs) have been identified from the shells of various Mollusca genera, including *Mytilus* (Gao et al., 2015; Liao et al., 2015), *Ostrea* (Zhang et al., 2012), *Pinctada* (Sarah et al., 2015), and *Perna* (Liao et al., 2019). The roles of PDCPs in shell formation remain a mystery. PDZ-binding proteins, such as TAZ (Transcriptional coactivator with the PDZ-binding motif), are known to play important roles in osteogenic differentiation and bone formation (Deng et al., 2008; Zhu et al., 2018), indicating the possible roles of the PDZ domain in biomineralization. In *M. coruscus*, PDCP-1 was identified from the myostracum, a shell layer that is secreted in an area of muscle attachment (Liao et al., 2015).

To explore the possible roles of PDCP-1 in biomineralization, PDCP-1 was recombinantly expressed, and the functions of this recombinant production were investigated. Further, the locations of PDCP-1 in the mantle, adductor muscle and shell surface as well as the possible protein partners of PDCP-1 were analyzed in this study. Our findings provide insight into the molecular mechanisms of PDCP-1 proteins associated with biomineralization.

## Materials and Methods

### Sequence Analysis and the Expression of the PDCP-1 Gene in *M. coruscus* Tissues

PDCP-1 was identified through transcriptome-proteome strategies in a previous work (Liao et al., 2015). The full-length cDNA sequence of PDCP-1 was screened out from the transcriptomic data of the *M. coruscus* mantle based on the protein fragment sequences from LC-MS/MS data. The cDNA sequence of PDCP-1 was further confirmed using PCR with primers “–CAACCACAACAACAACGACAAG-” (Forward primer) and “–TCTCCTCCTGACCATCCTGAA-” (Reverse primer) and was verified by PCR production sequencing. The PDCP-1 sequence was analyzed using conventional bioinformatic tools, including ORF Finder^1^, protein BLAST^2^, SignalP Server^3^, SMART domain prediction^4^, Phyre secondary structure prediction^5^, and SWISS MODEL tertiary structure prediction^6^.

Healthy adults of the mussel *M. coruscus* (with an average shell length of ∼7 cm) were collected from the coast of Zhoushan Island, China. These mussels were acclimated in a 300 L aquarium under laboratory conditions with a temperature of 20°C and salinity of 25‰ for 1 week before use. Mussels were fed daily with spirulina powder, and filtered seawater was changed for half the aquarium every day. After cryo-anaesthesia, the tissues (mantle, adductor muscle, foot, gill, blood, and gonad) were dissected from six adult individuals to examine the tissue distribution of the PDCP-1 gene. All tissue samples were immediately frozen in liquid nitrogen and stored at −80°C until RNA extraction. Total RNA was extracted by a traditional Trizol extraction method (Chomczynski, 1993). After extraction, RNA was examined for quality and concentration by a NanoDrop micro-spectrophotometer (Thermo, United States). The first strand of cDNA was synthesized using the PrimeScript^TM^ RT kit (Takara). The quantitative real-time PCR (qRT-PCR) analyses were performed with three independent replicates using SYBR^®^ Premix Ex Taq^TM^ (Takara) on a MX3000P Real-Time PCR System (Stratagene, United States). The specific primers derived from the sequence of PDCP-1 were designed and synthesized, including PDCP-1/F (GCGGAATTAAACCTTGGGAG) and PDCP-1/R (AGCGGGTGCTTGTCGTTG). The relative expression levels were measured using the 2^–Δ^ ^Ct^ method (Livak and Schmittgen, 2001) with β-actin and 18sRNA as internal references. The cycling conditions were: 3 min at 94°C followed by 40 cycles of 94°C for 10 s, 60°C for 40 s, and 72°C for 30 s.

### *In situ* Hybridization of PDCP-1

To determine the location of PDCP-1 mRNA expression in the mantle and the adductor muscle, *in situ* hybridization was performed. The fixed tissues were dehydrated through an ethanol series and then subjected to a xylene bath prior to paraffin embedding. Paraffin blocks were sectioned at 5 μm thickness. After treatment with proteinase K at 37°C for 20 min, the sectioned tissues were washed by a freshly prepared 0.1 M glycine solution for 1 min and PBS for 2 min. The tissues were immediately fixed in 4% paraformaldehyde for 10 min, followed by incubation at 65°C with FAM-labeled probe (5′-FAM-ACCAUUGAUAGCAGUAAGCACAUCUGUC-3′) for 48 h. The sectioned tissues were washed in formamide-4X SSC at 60°C. Signals were visualized with a substrate, 4’,6-diamidino-2-phenylindole (DAPI) reagent.

### Expression and Purification of Recombinant PDCP-1

The PDCP-1 gene encoding the mature peptide was codon-optimized and synthesized for an *E. coli* expression system. *Nco*I and *Xho*I restriction sites were attached to the 5′ and 3′ ends of the optimized sequence, respectively. The synthetic codon-optimized genes were excised by *Nco*I and *Xho*I digestion and ligated into a *pET/28*α expression vector. The construct was designed to yield a recombinant protein product with a molecular weight (MW) of ∼80 kD, fused in frame with His_6_ tags.

The recombinant PDCP-1 was expressed in *E. coli* strain BL21 (DE3). *E. coli* cells containing *rPDCP-1/pET/28*α were grown in Luria–Bertani (LB) liquid medium (Sangon Biotech, Shanghai, China) with 10 μg/mL kanamycin at 37°C. Isopropyl-D-thiogalactopyranoside (IPTG) with a final concentration of 1 mM was used for induction of recombinant PDCP-1. The induced cells were grown for 4 h, harvested and centrifuged at 1,000 × g for 15 min at 4°C, and the pellets were stored at −20°C for further use.

The cell pellets were dissolved in ice-cold lysis buffer (10 mM imidazole, 50 mM PBS, 100 mM NaCl, 1 M EDTA, pH 8.0) and homogenized using a sonicator at 4°C. The inclusion bodies were harvested by centrifugation (8,000 × g, 10 min, 4°C) and dissolved in a buffer (10 mM imidazole, 8 M urea, 100 mM NaCl, 100 mM PBS, pH 8.0) overnight at 4°C. Using a Ni-NTA column (Sangon Biotech, Shanghai, China), rPDCP-1 was purified by elution buffer (300 mM imidazole, 8 M urea, 100 mM NaCl, 100 mM PBS, pH 8.0). Isolated rPDCP-1 was refolded in a buffer containing the oxidized and reduced glutathione (GSH/GSSG) and dialyzed in graded concentrations of urea (0∼8 M) according to the protocol (Zhang et al., 2011).

After refolding, the rPDCP-1 was isolated by high-performance liquid chromatography (HPLC, Waters 650E, United States) with a reverse-phase C4 column (4.6 mm × 250 mm, 300 Å, Agilent). The eluted protein fraction from HPLC was lyophilized and stored at −20°C before use. SDS-PAGE was performed on a 12% polyacrylamide gel, and the protein bands were visualized using Coomassie Brilliant Blue R250.

### Functional Analysis of rPDCP-1

*In vitro* crystal growth experiments (Liang et al., 2015; Jiang et al., 2020) were performed to test the effects of rPDCP-1 on the morphology of calcite and aragonite crystals. The rPDCP-1 was incubated with a freshly prepared saturated solution of calcium carbonate (Yan et al., 2007) on a siliconized cover glass with or without magnesium chloride. Crystallization experiments were carried out with various concentrations of rPDCP-1 (10, 30, and 50 μg/mL). The morphology of calcium carbonate crystals induced by rPDCP-1 was observed by a Nova nano 450 (FEI) scanning electron microscopy (SEM) system. Polymorphism of calcium carbonate crystals was further identified by FTIR spectroscopy (Nicolet Nexus 670).

Crystal binding experiments were performed to test the interaction between rPDCP-1 and calcite or aragonite calcium carbonate. The calcite and aragonite calcium carbonate crystals were freshly prepared using the methods of Yan et al. (2007) and Jiang et al. (2020). rPDCP-1 was dissolved (1 mg/mL, as sample I) and incubated with calcite or aragonite crystals at room temperature for 1 h. After centrifugation (10,000 × g, 15 min), the supernatants were collected as sample II. The sediments were decalcified and centrifuged (10,000 × g, 15 min), and the supernatants were dialyzed (1 kDa cut-off) and used as sample III. Samples I∼III were each analyzed by SDS-PAGE.

The inhibition of rPDCP-1 on calcium carbonate precipitation was tested using the methods provided by Liang et al. (2015) and Jiang et al. (2020) with minor modifications. Briefly, 10 mM calcium chloride containing rPDCP-1 at various concentrations (10, 30, and 50 μg/mL) was dropped in a 96-well plate, and the plate was placed in a closed desiccator. Solid ammonium carbonate was added in the desiccator for calcium carbonate precipitation. The turbidity of the calcium chloride solution was monitored every minute for 10 min by measuring the absorbance at 630 nm with a micro-plate reader (Synergy H1, BioTec).

### Statistical Analysis

All data were expressed as the means ± SD (*n* = 3) of triplicate experiments. Tissues were used as a fixed factor, and replicates were used as a random factor. Homogeneity of variances and normality in the residual distributions were assessed by the Cochran test (Underwood, 1997) and Shapiro-Wilk normality test (Patrick, 1982), using the R GAD and stats packages. For the data that did not follow a normal distribution, the non-parametric Kruskal-Wallis test (Myles and Douglas, 1973) was used to assess the significant differences among groups. Further, following a significant Kruskal-Wallis test, a *post hoc* test, Dunn’s multiple comparison test (Dunn, 1964), was used to compare differences between groups. All statistical analysis was performed in the R environment (version 3.6.0).

### Polyclonal Antibody Preparation and Immunohistochemistry Analysis

The purified rPDCP-1 was enriched and submitted to HuaAn Biotechnology Co., Ltd. (Hangzhou, China) to produce polyclonal antibodies. Briefly, polyclonal antibodies were prepared by immunizing New Zealand rabbits with 0.5 mL of rPDCP-1 (1 mg/mL) with an equal volume of complete adjuvant. Three booster injections each containing 0.5 mL of rPDCP-1 (1 mg/mL) plus incomplete adjuvant were subsequently given at 1 week intervals. The antiserum was collected through the carotid artery 7 days after the last immunization and further purified by a protein A/G column.

The specificity of the antibodies was assayed by western blotting with soluble and insoluble matrices extracted from three layers (nacre, myostracum, and fibrous prism) of *M. coruscus* shell. Shell matrices were extracted from the shell as described previously (Liao et al., 2015) and isolated by SDS-PAGE. The PAGE gel was then transblotted onto a PVDF membrane. The anti-rPDCP-1 polyclonal antibody (1:2,000) was used as the primary antibody, and horseradish peroxidase-labeled goat anti-rabbit IgG (1:10,000; HuaAn Biotechnology Co., Ltd.) was used as the secondary antibody. Blots were visualized using 3, 3′, 5, 5′-tetramethylbenzidine stabilized substrate. The protein band intensities of this blot were measured and quantified using ImageJ software, and changes in the PDCP-1 levels were normalized to β-actin.

To determine the tissue distribution of naturally occurring PDCP-1, the tissues (mantle and adductor muscle) of *M. coruscus* were collected and fixed in 10% formaldehyde overnight and then dehydrated through ascending grades of ethanol. Sections (4 μm) were cut by a microtome and collected on coated slides for immunohistochemistry. After de-waxing and rehydration, the slides were incubated with the anti-rPDCP-1 antibody (1:200), supplemented with 1% BSA overnight at 37°C. The primary antibodies were detected using a peroxidase-conjugated antibody against rabbit IgG and stained by DAB solution. The sections were examined and photographed using a microscope (DFC450C, Leica, Germany).

Using the anti-rPDCP-1 antibody, the location of PDCP-1 on the shell inner surface was determined by immunofluorescence according to the technique described by Kong et al. (2018) and Jiang et al. (2020) with modification. Briefly, the shell was cut into pieces ∼1 cm^2^ containing the AMS. The shell samples were washed and sonicated in 5% NaOH to remove the remaining adductor muscles and organic contaminants on the shell surface, followed by soaking the sample in a stationary liquid containing 10% formaldehyde and 4% formic acid for 24 h. Then, the shell pieces were cleaned and treated with 0.25% Triton X-100 for 30 min and blocked with 10% negative goat serum for 1 h at 37°C. Immunostaining was performed by incubating the shell samples with anti-rPDCP-1 polyclonal antibody (1:50) and Alex488-conjugated goat-anti-rabbit antibody (1:500). Stained sections were examined with a fluorescence microscope (DMIL LED FLUO, Leica, Germany) equipped with a DFC450C digital imaging system (Leica, Germany). The deproteinized shell samples treated by 20% NaOH at 60 for 1 h were used as negative controls.

### His-Tag Affinity Pull-Down

Ni-NTA beads (Sangon, China) were used to binding the rPDCP-1 protein containing the His_6_ tag. After binding with rPDCP-1, the Ni-NTA beads were washed with binding buffer (20 mM Tris-HCl, 150 mM NaCl, 10 mM imidazole, pH 8.0), incubated with total proteins extracted from the shell of *M. coruscus* (Liao et al., 2015) for 4 h at 4°C, and then washed with elution buffer (20 mM Tris-HCl, 300 mM NaCl, 300 mM imidazole, pH 8.0). Eluted protein samples were analyzed by LC-MS/MS after digestion by trypsin. LC-MS/MS experiments were performed on a Q Exactive Plus MS coupled with an Easy nLC (Thermo Scientific). The MS data were analyzed using MaxQuant software (version 1.6.1.0.) and searched against the mantle transcriptome database of *M. coruscus* (Accession: SRX792025) (Liao et al., 2015). The database search results were filtered and exported with a < 1% false discovery rate (FDR) at the peptide-spectrum-matched level and protein level, respectively.

### Biolayer Interferometry and Co-immunofluorescence

The binding of rPDCP-1 with actin was measured by Biolayer Interferometry (BLI) on an Octet RED BLI (Pall ForteBio) at 25°C (Petersen, 2017; Jiang et al., 2020). First, rPDCP-1 (1 mM) was dissolved in PBS buffer (pH 7.4) containing 0.05% (v/v) Tween 20 and 0.1% (v/v) BSA and then loaded onto APS biosensors (aminopropylsilane), which were previously coated with 10 μg/mL actin, and then was incubated and measured. The procedure was as follows: 60 s for baseline1, 900 s for loading, 300 s for baseline2, 300 s for association, and 120 s for dissociation. The affinity (K_D_) was determined by BLI using an Octet RED96E instrument (ForteBio) (Ekiert et al., 2012).

The location and the interaction of PDCP-1 with actin on the shell inner surface were determined by co-immunofluorescence as described previously (Hiong et al., 2016; Jiang et al., 2020) with minor modification. Briefly, the shell was cut into pieces of approximately 1 cm^2^ containing the AMS. The samples were washed and sonicated in 5% NaOH to remove remaining adductor muscle and organic contaminants from the shell surface. Then, the shell pieces were cleaned and treated with 0.25% Triton X-100 for 30 min and blocked with 10% negative goat serum for 1 h at 37°C. Immunostaining was performed by incubating the shell samples with anti-rPDCP-1 polyclonal antibody (1:500) and Alexa 488-conjugated goat-anti-rabbit antibody (1:5,000), or mouse anti-actin monoclonal antibody and Alexa 555-conjugated goat-anti-mouse antibody (1:5,000). The stained sections were examined with a fluorescence microscope (DMIL LED FLUO, Leica, Germany) equipped with a DFC450C digital imaging system (Leica, Germany). Deproteinized shell samples treated with 20% NaOH at 60°C for 1 h were used as negative controls.

## Results

### Features of the PDCP-1 Sequence

Bioinformatics analysis revealed that the full length of native PDCP-1 cDNA was 1,820 bp, and the open reading frame (1,788 bp) encodes a 595 amino acid (AA) precursor (Supplementary Figure 1), with the theoretical molecular weight of 67.7 kDa and the isoelectric point (*pI*) of 9.37. A PDZ domain (residues 20–92) and a ZM domain (residues 497∼522) were detected in the PDCP-1 precursor (Supplementary Figure 2). The secondary structure of PDCP-1 is composed of 18% α-helix, 8% β-sheet, and 78% disorder (Supplementary Figure 2). The predicted tertiary structure of PDCP-1 is composed of five β-sheets and two α-helices (Supplementary Figure 2), resembling the standard PDLIM proteins from humans (Elkins et al., 2010). Protein Blast revealed that PDCP-1 shared low sequence identity with AIM3-like (altered inheritance of mitochondria protein 3-like) and INTS3 (integrator complex subunit 3) from *Crassostrea gigas* and with PDLIM3 from *Mizuhopecten yessoensis*. A total of 21 representative homologs were selected to build a phylogenetic tree. In the phylogenetic tree, four conspicuous branches, namely, membrane-associated guanylate kinase, PDLIM proteins, extensin-like proteins, and INTS branches, were presented (Supplementary Figure 3). PDCP-1 is located at an independent branch and is grouped closely with PDLIM3 from *Mizuhopecten yessoensis* (Supplementary Figure 3). The domain organization of PDCP-1 with its homologs from mollusks is shown in Supplementary Figure 4.

### Tissue Expression and *in situ* Hybridization

**FIGURE 1:**
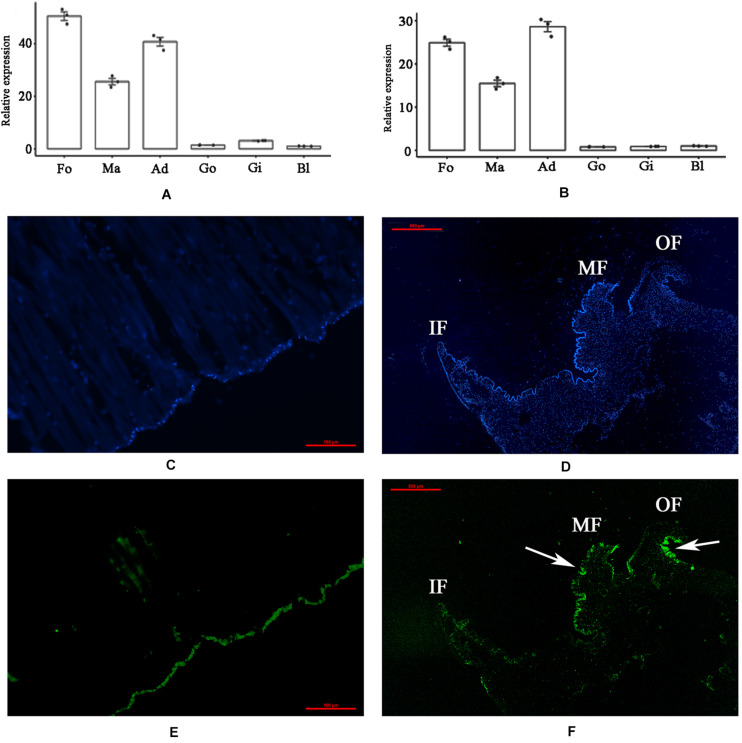
Tissue-specific expression and *in situ* hybridization of PDCP-1 in adductor muscle and mantle. Ma, mantle; Ad, adductor muscle; Fo, foot; Go, gonad; Gi, gill; Bl, blood. Values presented by means ± SD of three replicates. **(A)** tissue-specific expression level of PDCP-1 in various tissues using β-actin as reference gene; **(B)** tissue-specific expression level of PDCP-1 in various tissues using 18s RNA as reference gene; **(C)** adductor muscle of control group; **(D)** mantle of control group; **(E)** expression of PDCP-1 (green color) in adductor muscle; **(F)** expression of PDCP-1 (green color) in mantle. The scale bar, 100 μm for **(B,C)**, and 500 μm for **(D,E)**. IF, inner fold; MF, middle fold; OF, outer fold.

The tissue-specific expression of PDCP-1 was investigated by qRT-PCR. The mRNA transcript of PDCP-1 was detected in all six tested tissues, namely, the mantle, adductor muscle, gill, foot, blood and gonad. The highest expression level of PDCP-1 was observed in muscle-related tissues, including adductor muscle, foot, and mantle (Figures 1A,B). Using FAM-labeled PDCP-1-specific probes, strong signals were detected mainly at the edge of the middle fold and the outer fold of the mantle, as well as the bottom of the adductor muscle near the shell surface (Figures 1C–F).

### Recombinant Expression and Purification of PDCP-1

**FIGURE 2:**
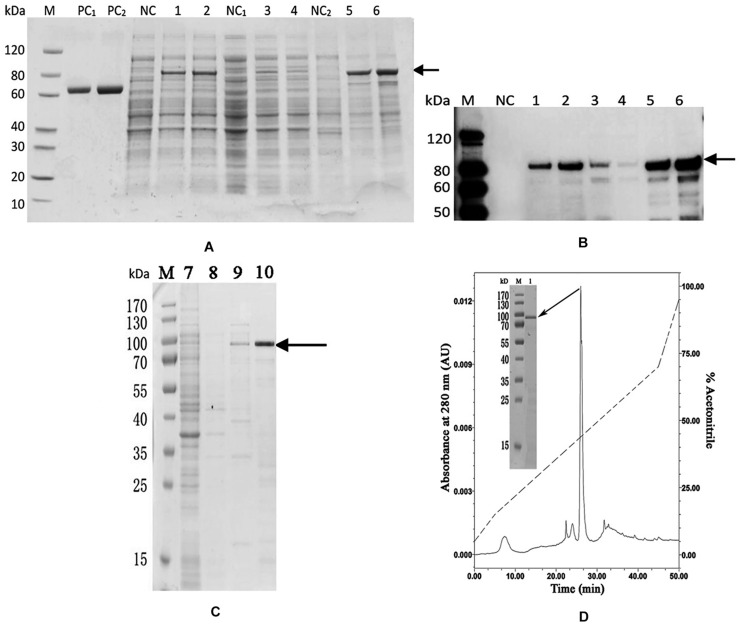
Recombinant expression and purification of rPDCP-1. **(A)** SDS-PAGE of recombinant expressed rPDCP-1; **(B)** Western Blot of rPDCP-1; **(C)** isolation of rPDCP-1 by Ni-NTA column; **(D)** HPLC purification of rPDCP-1 after refolding. Lane M, protein marker; Lane PC1, BSA(1 mg); Lane PC2, BSA(2 mg); Lane NC, cell lysate of negative control without IPTG induction; Lane 1, cell lysate with IPTG induction for 16 h at 15°C; Lane 2, cell lysate with IPTG induction for 4 h at 37°C; Lane NC1, the supernatant of cell lysate without IPTG induction; Lane 3, the supernatant of cell lysate with IPTG induction for 16 h at 15°C; Lane 4, the supernatant of cell lysate with IPTG induction for 4 h at 37°C; Lane NC2, the debris of cell lysate without IPTG induction; Lane 5, the debris of cell lysate with IPTG induction for 16 h at 15°C; Lane 6, the debris of cell lysate with IPTG induction for 4 h at 37°C; Lane 7, isolation of rPDCP-1 by Ni-column after sample loading and eluted by 10 mM imidazole; Lane 8, eluted rPDCP-1 by 30 mM imidazole; Lane 9, eluted rPDCP-1 by 100 mM imidazole; Lane 10, eluted rPDCP-1 by 300 mM imidazole. The protein band with ∼80 kDa (indicated by an arrow) corresponds to the rPDCP-1. The primary antibody for Western Blot is anti-His_6_ antibody (GenScript, Cat.No.A00186).

The codon optimized PDCP-1 gene was successfully expressed by a *pET-28/E. coli* expression system with IPTG induction. SDS-PAGE (12%) analysis revealed the presence of rPDCP-1 with the expected molecular weights (∼80 kD) of the fusion proteins (with His_6_ Tags) in the pellet fraction of *E. coli* lysates (Figure 2A), which were further confirmed by western blotting with anti-His-Tag antibody (Figure 2B). The expression levels of rPDCP-1 were estimated as 5 mg/L culture by calculating the OD value of the standard BSA band loaded in the SDS-PAGE gel (lanes PC1 and PC2 of Figure 2) and comparing this with the OD values of the bands from rPDCP-1 in the same gel. The recombinant His-tagged proteins were purified by affinity chromatography with a Ni-column from the pellet fraction of *E. coli* lysates (Figure 2C). Using a GSH/GSSH oxidation buffer containing urea, the recombinant proteins were refolded stepwise by dialysis against a series of urea concentrations from high to low. Refolded proteins were then purified by reverse-phase HPLC using a C4 column, resulting in high purity (Figure 2D).

### Functions of rPDCP-1

**FIGURE 3:**
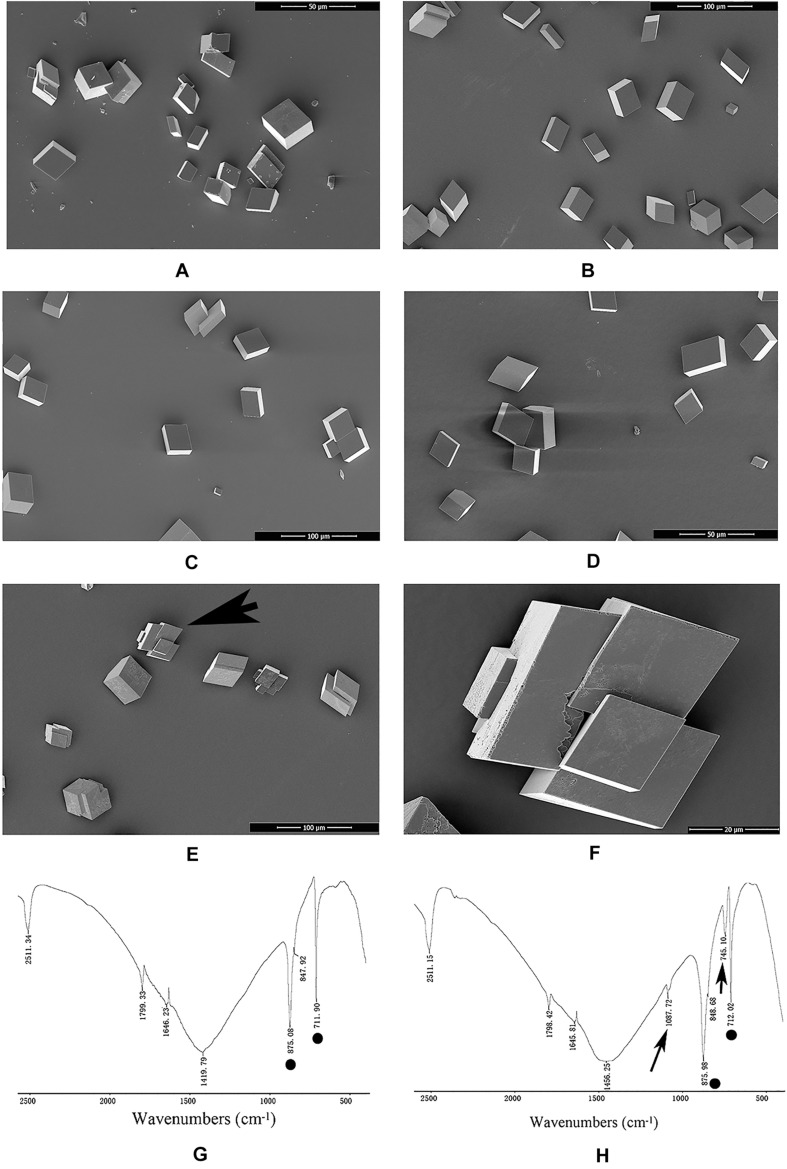
SEM images and FTIP spectra of *in vitro* calcite crystallization in the presence of rPDCP-1 at increasing concentrations. **(A)** control calcite crystals grown without induction; **(B)** calcite crystals grown with 50 μg/mL BSA; **(C)** crystals grown with 10 μg/mL rPDCP-1; **(D)** calcite crystals grown with 30 μg/mL rPDCP-1. **(E)** calcite crystals grown with 50 μg/mL rPDCP-1. **(F)** enlarged image of **(E)**; **(G)** FTIR spectrum of control calcite; **(H)** FTIR spectrum of calcite crystals induced by 50 μg/mL rPDCP-1. Arrows indicate the characteristic peaks of aragonite induced by rPDCP-1. The circles represent characteristic peaks of calcite. Scale bar: 50 μm for **(A,D)**, 100 μm for **(B,C,E)**, 20 μm for **(F)**.

**FIGURE 4:**
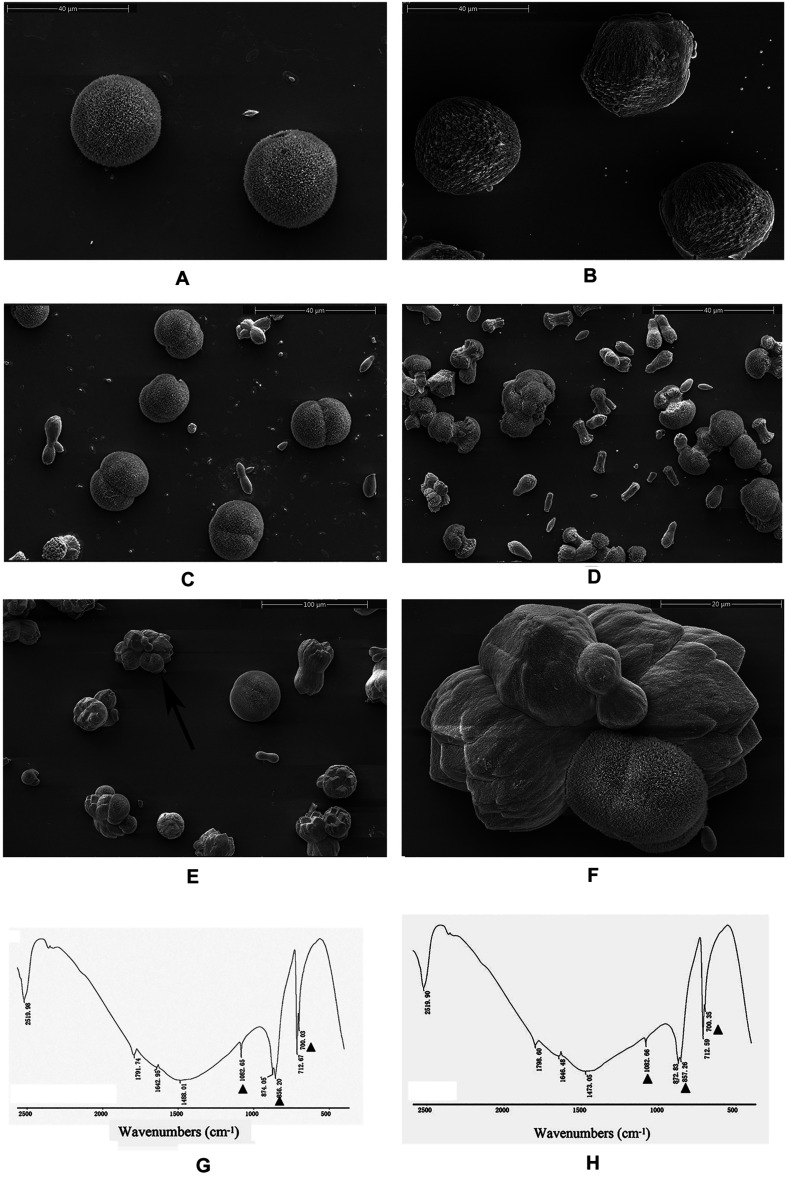
SEM images of *in vitro* aragonite crystallization in the presence of rPDCP-1 at increasing concentrations. **(A)** control aragonite crystals; **(B)** aragonite crystals grown with 50 μg/mL BSA; **(C)** aragonite crystals grown with 10 μg/mL rPDCP-1; **(D)** aragonite crystals grown with 30 μg/mL rPDCP-1; **(E)** aragonite crystals grown with 50 μg/mL rPDCP-1; **(F)** enlarged image of **(E)**; **(G)** FTIR spectrum of control aragonite; **(H)** FTIR spectrum of aragonite crystals induced by 50 μg/mL rPDCP-1. The triangles represent characteristic peaks of aragonite. Scale bar: 40 μm for **(A–D)**, 100 μm for **(E)**, 20 μm for **(F)**.

An *in vitro* calcium carbonate crystallization assay was performed, and the results revealed little effect of rPDCP-1 on the morphology of calcite crystals but significant effects on the morphology of aragonite crystals (Figures 3, 4). For the negative control calcite groups, the crystals were typical rhombohedra without or with induction with 50 μg/mL BSA (Figures 3A,B). While rPDCP-1 was added with increasing concentrations, the morphology of calcite crystals presented only weak changes at a high concentration (50 μg/mL) of rPDCP-1, and the induced crystals were observed as stacked cubes (Figures 3C–F). For the aragonite crystals, the rPDCP-1 showed significant effects on the crystal morphology. As shown in Figure 4, natural globular aragonite crystals were altered with the increasing concentration of rPDCP-1, from globular splitting to an irregular radial shape.

FTIR spectroscopy was used to characterize the polymorphs of induced crystals. As shown in Figure 3, the calcite crystals grown in the control experiments were shown to be calcite with specific peaks (Figure 3G), while the rPDCP-1-induced crystals showed extra aragonite specific peaks at 1087.72 cm^–1^ (Figure 3H). For the aragonite crystals, no changes in the polymorph were detected before and after rPDCP-1 addition (Figures 4G,H), indicating that rPDCP-1 has no effects on the polymorph of aragonite crystals.

**FIGURE 5:**
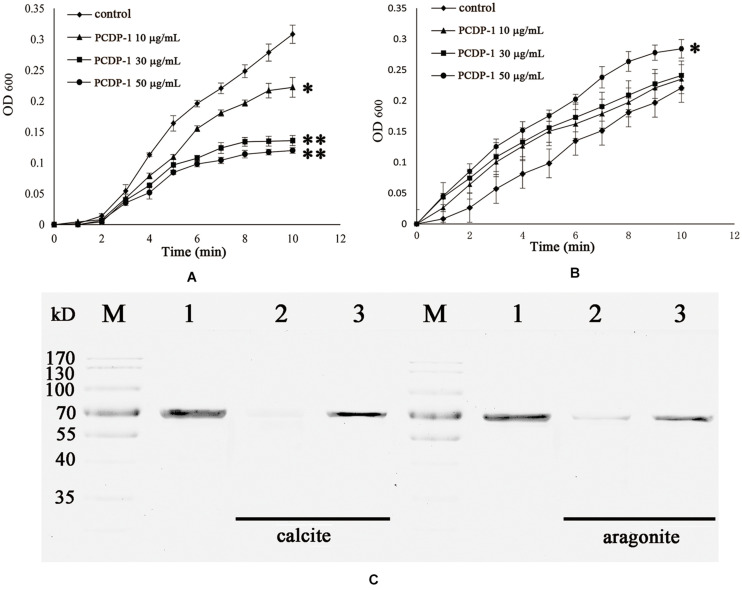
The crystallization rate inhibition of rPDCP-1 in calcite **(A)** and aragonite **(B)**, and the binding ability of rPDCP-1 with calcite and aragonite **(C)** respectively. The Data of **(A,B)** are represented by mean ± SD (*n* = 3). ^∗^*p* < 0.05; ^∗∗^*p* < 0.01. Lane M, protein marker; lane 1, the control rPDCP-1; Lane 2, the supernatant of the solution after that the rPDCP-1 was precipitated by CaCO_3_ crystals; Lane 3, the rPDCP-1 released from the precipitate of CaCO_3_ crystals.

The crystallization rate of calcium carbonate was measured by the absorbance at 630 nm. As shown in Figure 5, rPDCP-1 showed significant inhibition of the crystallization rate of calcite crystals in a dosage-dependent manner, and the highest absorbance values (at 50 μg/mL rPDCP-1) were no more than 0.2 (Figure 5A), compared with 0.3 for the control group. For the crystallization rate of aragonite, the rPDCP-1 showed slight promotion effects at 50 μg/mL, with the highest OD_630_ value of 0.29, compared with 0.22 for the control group (Figure 5B).

SDS-PAGE was used to detect the possible interaction between rPDCP-1 and calcium carbonate crystals. As shown in Figure 5C, lanes 1, 2, and 3 represent the pure rPDCP-1 solution, the supernatant of the rPDCP-1 solution after precipitation by calcium carbonate crystals, and the rPDCP-1 released from the precipitate of calcium carbonate crystals, respectively. For calcite crystal binding experiments, the absence of the rPDCP-1 band in lane 2 revealed the binding of rPDCP-1 with the insoluble calcite crystals, which was further confirmed by the reappearance of the protein band in lane 3. For the aragonite crystal binding experiment, the protein band of rPDCP-1 can be observed in both lane 2 and lane 3, suggesting that the binding of rPDCP-1 with aragonite crystals is weaker than that with calcite crystals.

### Localization of PDCP-1 in the Shell and Tissues of *M. coruscus*

**FIGURE 6:**
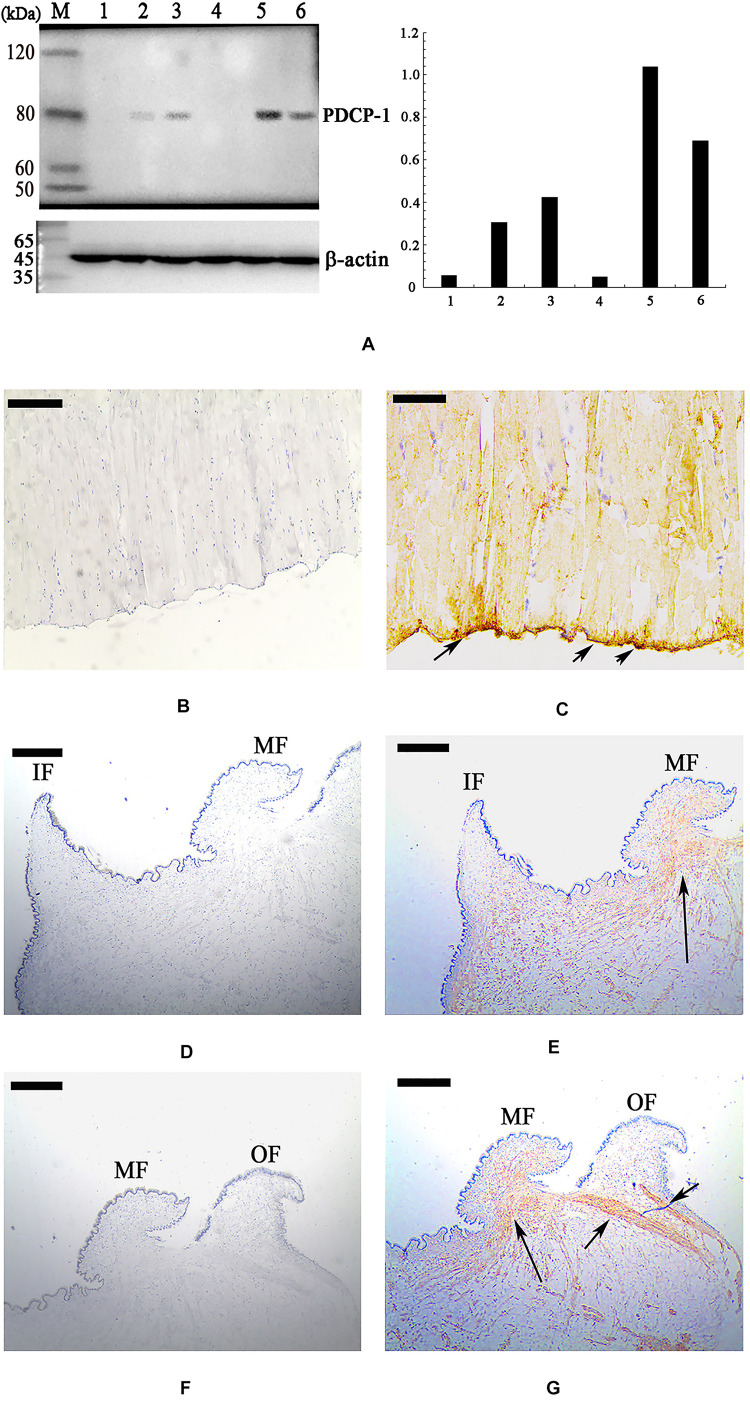
Western blot and immunohistochemistry analysis of PDCP-1. **(A)** Western blotting by anti-rPDCP-1 antibody in shell matrices (right panel) and the protein band intensity of PDCP-1 expressed as a bar diagram as a ratio, relative to beta-actin (left panel). M, protein marker; 1, acid-soluble fraction from the fibrous prismatic layer; 2, acid-soluble fraction from the nacre layer; 3, acid-soluble fraction from the myostracum layer; 4, acid-insoluble fraction from the fibrous prismatic layer; 5, acid-insoluble fraction from the myostracum layer; 6, acid-insoluble fraction from the nacre layer; **(B)** immunohistochemistry analysis with the control group of adductor muscle performed using only second antibody showed no significant signals; **(C)** detection of PDCP-1 in the adductor muscle and the positive signals are indicated by arrows; **(D)** the control group of mantle, showing the inner and the middle folds; **(E)** detection of PDCP-1 in the mantle and the positive signal is indicated by arrows; **(F)** the control group of mantle, showing the middle and the outer folds; **(G)** detection of PDCP-1 in the mantle and the positive signals are indicated by arrows. The scale bar, 50 μm.

Using the polyclonal anti-rCLP antibody prepared in this study, the location of PDCP-1 in the three shell layers was assessed using western blotting. As shown in Figure 6A (right panel), PDCP-1 was detected with the expected MW in both the acid-soluble and the acid-insoluble matrices from the myostracum layer and the nacre layer. These values are presented as ratios, compared to β-actin in bar diagrams (Figure 6A, left panel). The highest value is presented at the acid-insoluble matrices of the myostracum layer, followed by the acid-insoluble matrices of the nacre layer, the acid-soluble matrices of the myostracum layer, and the acid-soluble matrices of the myostracum layer.

Immunohistochemistry analysis revealed that PDCP-1 was expressed mainly at the bottom of the posterior adductor muscle, which was connected with the shell surface at the shell muscle scar (Figures 6B,C). In addition, the expression of PDCP-1 in the mantle observed to have weak signal inside the mantle under the middle fold and outer fold (Figures 6D–G).

### The Interaction Network of PDCP-1 in the Shell

**FIGURE 7:**
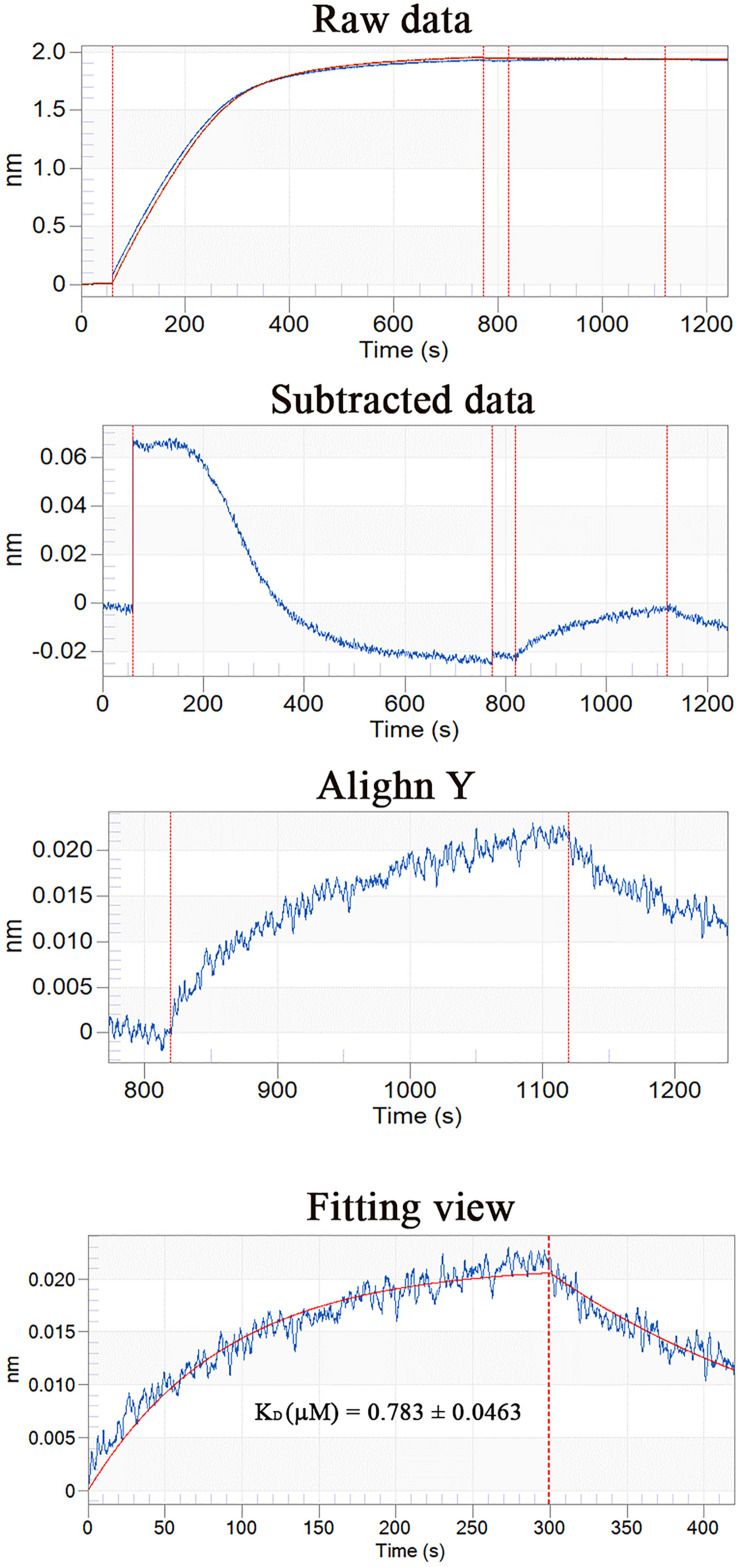
BLI curves (from the raw data to the final fitting view) for the binding of rPDCP-1 to biosensors coated with actin.

Using Ni-coupled pull-down technology, rPDCP-1 with His_6_ tag was used as bait, and protein partners were pulled from the shell matrices. After LC-MS/MS analysis, a set of 19 proteins was identified with FDR < 0.01 and matched unique peptides more than 2, including PDCP-1 itself (Table 1). The MS proteomics data have been deposited to the ProteomeXchange Consortium^7^ via the iProX partner repository (Ma et al., 2019) with the dataset identifier PXD017074. The main localizations and functions of these pulled proteins were cytoplasm (PDZ domain-containing protein, transgelin, whirlin, dehydrogenase, histone, and ubiquitin), cytoskeleton (tubulin and actin), biomineralization (shell mytilin, SD-rich protein, SK-rich protein, EP-protein, and alanine and glycine-rich protein), and others (protein Shroom 2, KAR9-domain-containing protein, ribosomal protein, and HSP90). Of these proteins, the binding of actin with rPDCP-1 was further measured by BLI and the raw data, the subtracted data, the aligned data, and the final fitting view are listed in Figure 7. The K_*D*_ of rPDCP-1 with actin was calculated by Fortebio Data Analysis software as 0.783 ± 0.0463 μM.

**TABLE 1:** LC-MS/MS identification of the protein partners of the rPDCP-1 from the matrices extracted from the shell of *M. coruscus* by affinity adsorption using Ni-column pull down.

**Protein IDs**	**Homology name (species)**	**Query cover**	***E*-value**	**Sequence identity**	**Accession**	**Unique peptides**	**Sequence coverage (%)**	**Score**	**Sequential features**
CL4409.Contig1	Collagen-like protein-2 (*Mytilus coruscus)*	100%	4.00E-36	100.00%	AKS48142.1	15	80.1	323.31	VWA (SM000327)
CL1310.Contig2	PDZ domain-containing protein-1 (*Mytilus coruscus*)	100%	1.00E-112	100.00%	AKS48171.1	10	32.9	323.31	Gln 22.5%
CL7857.Contig1	Shell mytilin-1 (*Mytilus coruscus*)	100%	2.00E-134	100.00%	AKI87978.1	8	57.5	323.31	Signal peptide (1-20); Leu 9.9%
Unigene32537	HSP90 (*Mytilus coruscus*)	100%	0	100.00%	ALL27016.1	6	9.4	63.982	HATPase_c (SM000387); Pfam:HSP90(PF00183)
CL228.Contig1	Tubulin beta-4B chain isoform X1 (*Echinops telfairi*)	96%	0	98.83%	XP_004702931.1	6	17.3	51.527	Tubulin(SM000864); Tubulin_C(SM000865)
CL7444.Contig2	Transgelin-like protein-3 (*Mytilus coruscus*)	97%	1.00E-53	96.93%	AKS48154.1	5	47.2	89.052	CH(SM000033)
CL5847.Contig1	SD-rich protein-1 (*Mytilus coruscus*)	100%	0	100.00%	AKS48139.1	5	19	72.238	Internal repeat 1 (1–24), internal repeat 1(27–50), Ser 17.8%
Unigene68573	Whirlin (*Mytilus coruscus*)	100%	5.00E-34	100.00%	QGA67049.1	5	39	53.277	PDZ(SM000228)
Unigene66132	SK-rich protein-1 (*Mytilus coruscus*)	100%	1.00E-121	100.00%	AKS48143.1	3	14.4	33.995	Ser 22.4%
CL28.Contig6	Tubulin alpha-1C chain (*Taeniopygia guttata*)	99%	0	91.52%	XP_030115314.1	3	11.6	31.83	Tubulin(SM000864); Tubulin_C(SM000865)
Unigene12026	Actin,cytoplasmic (*Stylophora pistillata*)	100%	0	97.61%	PFX22595.1	2	25.8	113.37	ACTIN(SM000268)
Unigene66002	ubiquitinCvariant (*Homo sapiens*)	100%	3.00E-32	100.00%	OLQ17059.1	2	32.5	59.83	UBQ (SM000213)
CL6812.Contig2	EP-protein-1 (*Mytilus coruscus*)	100%	3.00E-95	100.00%	AKS48159.1	2	5.9	14.279	Ser 22.4%
Unigene69724	30S ribosomal protein S11 (*Alphaproteobacteria bacterium RBG_16_64_48*)	90%	1.00E-67	36.30%	OFW72110.1	2	15.8	14.075	Pfam:Ribosomal_S11(PF00411)
Unigene13723	Glyceraldehyde-3-phosphate dehydrogenase GAPCP2 (*Spatholobus suberectus*)	99%	0	71.64%	TKY49434.1	2	7.8	13.564	Gp_dh_N(SM000846); Pfam:Gp_dh_C(PF02800)
CL6608.Contig1	KAR9-domain-containing protein (*Aspergillus costaricaensis CBS 115574*)	98%	7.00E-04	18.18%	XP_025541145.1	2	31.4	13.083	Pro 32.5%
Unigene69727	Alanine and glycine-rich protein(*Mytilus californianus*)	41%	1	89.29%	P86857.1		17.6	12.297	Gly 41.2%
CL6519.Contig3	protein Shroom2 (*Chrysochloris asiatica*)	33%	9.00E-118	31.55%	XP_006835763.1	2	1.3	11.66	Pfam:ASD2(PF08687)
Unigene72302	Histone H2B (*Mytilus galloprovincialis*)	100%	1.00E-39	100%	AAP94644.1	2	13	11.455	H2B(SM000427)

**FIGURE 8:**
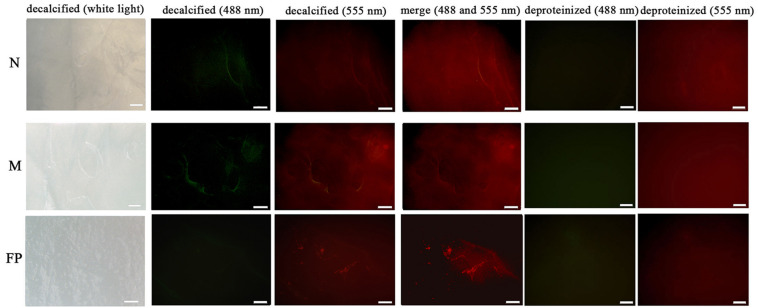
Immunofluorescence location of native PDCP-1 (green at 488 nm) and actin (red at 555 nm) on the surface of decalcified shell samples of *M. coruscus*, and the deproteinized shell samples were used as negative controls. N, nacre; M, myostracum; FP, fibrous prism. The scale bar, 100 μm.

The microstructural location of both PDCP-1 and actin on the shell surface was detected by double-labeling co-immunofluorescence (Figure 8). After decalcification, an organic membrane was present on the shell surface with different textures of the myostracum, nacre, and fibrous prism layers (Figure 8). The signal of both PDCP-1 (green) and actin (red) was detected on the myostracum and the nacre layer of the decalcified shell surface. Most of the PDCP-1 signal and the actin signal presented at the same region with the overlap model, suggesting an interaction between these two proteins (Figure 8). No PDCP-1 signal was detected on the fibrous prismatic layer, but a weak actin signal was observed in this layer. For those deproteinated shell samples, no signal from PDCP-1 or actin was detected (Figure 8).

## Discussion

SMPs have been reported as the main organic components that play crucial roles in *Mollusca* shell formation. More than 1,000 SMPs with various structures have been identified from many mollusk shells, and most of these SMPs were identified by a proteomic approach. However, only a few SMPs have been characterized individually to date because of the extremely small amounts of the matrix proteins in the shell and the difficulty in obtaining sufficient protein samples. In this study, PDCP-1, a Gln-rich shell protein with PDZ domain and ZM domain, was recombinantly expressed, and the function and the location of rPDCP-1 were determined to identify the possible mechanisms of this protein in shell formation.

In mollusks, the proteins containing the PDZ domain can be divided into four groups according to the domain organization based on the comparative results of domain organization. PDCP-1 belongs to group I, with PDLIM3 of *M. yessoensis*, extensin-like protein and trithorax group proteins from *Aplysia californica* in the same group. The members of this group have been reported with functions in actin-binding (Xia et al., 1997), cell wall construction (Rubinstein et al., 1995), and DNA-binding (Treisman et al., 1997), indicating diverse interactions of these proteins during their functions. However, the roles of PDCPs in biomineralization are still unknown, though various PDCPs, including PDZ/ZM and PDZ/LIM domain containing proteins, were identified previously from mollusk shells (Zhang et al., 2012; Gao et al., 2015; Liao et al., 2015; Sarah et al., 2015; Liao et al., 2019). PDCP-1 contains abundant Gln (18.2%) and repetitive “-QQQP(Y)Q(R)-” motifs in its sequence. SMPs rich in Gln have been found in various Mollusca shells, such as the pearl oyster *Pinctada margaritifera* (Berland et al., 2011), the gastropod *Lottia* (Marie et al., 2013), and the freshwater pearl mussels *Elliptio complanata* and *Villosa lienosa* (Marie et al., 2017). Interestingly, vertebrate teeth also contain Gln-rich proteins, which are believed to interact with calcium ions and regulate tooth mineralization (Kawasaki et al., 2009). In parallel, it has been proposed that numerous Gln residues are implicated in the tensile mechanical properties of various silk-like fibroins (Catesy et al., 2001). In addition, PDCP-1 contains 121 (20.3%) charged amino acids in its sequence. The charged amino acids of SMPs were reported to play important roles in biomineralization, either by forming a “calcium bridge” with calcium ions or interacting with negatively charged carbonate ions (Addadi and Weiner, 1985; Suzuki and Nagasawa, 2013). The abundant Gln, together with the charged amino acids of PDCP-1, indicates the function of this protein in biomineralization.

In *M. coruscus*, the higher expression level of the PDCP-1 gene in the adductor muscle indicates the potential function of PDCP-1 in this tissue, especially considering the roles of the PDZ domain in actinin binding (Liao et al., 2016). In addition, the higher expression level of PDCP-1 in foot and mantle indicates the possible roles of this protein in muscle attraction, as well as in bioadhesion and biomineralization, considering that the foot and the mantle are responsible for byssal adhesion (Rees et al., 2019) and shell formation (Wang et al., 2017), respectively. The expression of PDCP-1 was further detected with the location at the epidermis of the outer fold and the middle fold of the mantle by *in situ* hybridization experiments, implying the functions of PDCP-1 in prismatic layers (Addadi and Weiner, 1986; Shen et al., 1997). However, immunohistochemistry analysis using the anti-rPDCP-1 antibody revealed that PDCP-1 was present inside the mantle tissue other than at the edge. Therefore, we speculated that PDCP-1 may be secreted from the edge of the mantle and transported *via* unknown mechanisms into two places, the internal mantle (for the contraction of mantle muscle) and the shell (for controlling the crystallization of CaCO3). In addition, *in situ* hybridization and immunohistochemistry analysis revealed the location of PDCP-1 at the bottom of the adductor muscle. In *M. coruscus*, the adductor muscle is connected with the shell *via* organic membranes at the bottom of the adductor muscle, as well as on the surface of the shell myostracum layer (Liao et al., 2015). The strong signal of PDCP-1 at the bottom of the adductor muscle, detected by either *in situ* hybridization or immunohistochemistry, suggested the possible function of PDCP-1 in muscle-shell attachment.

In this study, PDCP-1 was successfully expressed using codon optimization strategy and a prokaryotic recombinant expression system. The recombinant PDCP-1 (rPDCP-1) showed significant alteration of the morphology of aragonite and the polymorph of calcite, indicating selective functions of rPDCP-1 for different calcium carbonate crystals. In addition, rPDCP-1 showed inhibition of the crystallization rate for calcite and promotion of aragonite, suggesting that rPDCP-1 may have the ability to accelerate the formation of aragonite crystals in *Mytilus* shell. Interestingly, rPDCP-1 was observed to have binding abilities for both calcite and aragonite crystals, indicating an interaction of rPDCP-1 with calcium carbonate crystals. As reported previously, SMPs can bind to the surface of calcium carbonate crystals, decreasing (or increasing) the growth of calcium carbonate crystals accordingly, and finally induce crystals forming different morphologies and polymorphs (Suzuki and Nagasawa, 2013). This viewpoint can partially explain the effects of PDCP-1 on calcite crystallization but still needs to be studied in more detail. According to the different functions of PDCP-1 in calcite vs. aragonite, we speculate that PDCP-1 plays important roles in the formation of aragonite via the promotion of aragonite crystallization and the transformation of calcite to aragonite. This speculation is based on the primary distribution of PDCP-1 in the myostracum layer with aragonite composition (Liao et al., 2015) and on previously proposed hypotheses (Addadi and Weiner, 1986; Rees et al., 2019), in which the formation of aragonite shell layers may result from the transformation of calcite crystals under the guidance of SMPs.

As highlighted previously, assembly of a biochemical framework is essential for shell formation (Nudelman et al., 2006; Evans, 2019), and the formation of the framework layer in mollusk shells relies on specific matrix-forming proteins, such as silk-like proteins and chitin-binding proteins (Evans, 2019). In this study, 19 proteins were identified by the pull-down technique combined with LC-MS/MS analysis, providing a set of candidate proteins that interact with PDCP-1. Most of the proteins that were pulled down had been identified from the shell proteome of *M. coruscus* (Liao et al., 2015) or other mollusks (Zhang et al., 2012; Gao et al., 2015; Sarah et al., 2015; Liao et al., 2019). We noted that PDCP-1 contains abundant (78%) intrinsically disordered sequence regions, indicating that PDCP-1 is conformationally unstable. Intrinsically disordered regions of SMPs were identified as signature sequence traits for shell matrix assembly and mineralization (Evans, 2012). Thus we proposed that PDCP-1 may use these intrinsically disordered regions to interact with either other SMPs and/or the mineral phase.

Among the identified proteins, collagen-like protein-2 (CLP-2) and whirlin were characterized recently from *M. coruscus* shell with biomineralization-related functions (Sun et al., 2020; Jiang et al., 2020). CLP-2 is a vWA (von Willebrand factor type A) domain-containing SMP, and the vWA domain was reported to show accessibility for intermolecular interactions during mollusk shell formation (Chang and Evans, 2015). Whirlin is a PDZ domain-containing SMP, and the interaction of PDZ-PDZ was previously confirmed (Sotelo et al., 2015; Murciano-Calles et al., 2019). In addition, two Ser-rich SMPs (SD-rich protein-1 and SK-rich protein-1) were identified from the pulled down proteins. Ser is the key amino acid of the PDZ-binding motif (Tandon et al., 2007; Bidoia et al., 2010), and the phosphorylation of Ser/Thr has a profound effect on PDZ binding (Hegedüs et al., 2003). HSP90 is another protein partner of PDCP-1, and previous research revealed the HSP90/PDZ interaction in human breast cancer cells (Jeong et al., 2019). These results suggested that PDCP-1 may be involved in an interaction network within the shell via the PDZ domain. Although more detailed studies are necessary for exploring the real interaction between PDCP-1 and the identified protein partners, the pull-down results of PDCP-1 provide a clue for studying shell protein–protein interactions and for any attempts to understand the supramolecular chemistry contributing to shell formation.

The interaction of the PDZ domain with actin was proposed in previous literature (Hildebrand and Soriano, 1999). Therefore, the interaction affinity between PDCP-1 and actin was measured by BLI, which revealed a K_D_ of ∼0.78 μM for PDCP-1 binding to actin. Double-labeling co-immunofluorescence further revealed the location of PDCP-1 together with the actin on the shell surface and confirmed the interaction of PDCP-1 with the actin at the nacre and the myostracum layer. The PDZ/actin interaction may be important for shell formation and the attachment between adductor muscle-myostracum, considering that abundant actin was identified from *Mytilus* shell (Gao et al., 2015; Liao et al., 2015), and the actin is also the main component of adductor muscle (Hue et al., 1989).

In summary, PDCP-1 is a novel shell matrix protein with PDZ and ZM domains. Recombinantly expressed PDCP-1 altered the morphology, polymorphism, and crystallization rate of calcium carbonate crystals. The specific location of PDCP-1 in the mantle, adductor muscle, and shell surface supports the proposal that this protein plays a role in biomineralization and muscle-shell attachment. We are fully aware that the functional analysis of a single shell matrix protein is not sufficient to provide an explanation for the whole process of shell fabrication. However, we consider that the characterization of biomineralization-related proteins one-by-one will provide the complete biochemical framework required to precisely analyze the formation process of the shell.

## Data Availability Statement

All datasets presented in this study are included in the article/Supplementary Material.

## Author Contributions

ZL and QS designed the experiments and wrote the manuscript. QS and YJ analyzed the data. QS, YJ, HX, MF, and XZ carried out the experiments. XY contributed the materials. All authors carried out the revision of the manuscript and gave final approval for publication.

## Conflict of Interest

The authors declare that the research was conducted in the absence of any commercial or financial relationships that could be construed as a potential conflict of interest.

## Footnotes

1 https://www.ncbi.nlm.nih.gov/orffinder

2 https://blast.ncbi.nlm.nih.gov/Blast.cgi

3 http://www.cbs.dtu.dk/services/SignalP/

4 http://smart.embl-heidelberg.de/

5 http://www.sbg.bio.ic.ac.uk/phyre2

6 https://swissmodel.expasy.org/interactive

7 http://proteomecentral.proteomexchange.org

## Supplementary Material

Supplementary Figure 1 Alignment of the cDNA with the deduced amino acid sequence of PDCP-1 (GenBank AKS48142.1). The repetitive “-QQQP(Y)Q(R)” was denoted by frames. *Represents the termination codon.

Supplementary Figure 2 Structural features of PDCP-1. **(A)** The secondary structure of PDCP-1 predicted by Phyre. The regions adopting putative α-helix and β-sheet are represented as spiral and arrow, respectively. **(B)** Domains of PDCP-1 were predicted by SMART and one PDZ domain and on ZM domain were showed. C: the spatial structure of PDCP-1 predicted by SWISS-MODEL. Model 1∼3 represent the predicted structures with highest score using 1v5l.1.A, 2uzc.5.A and 2q3g.1.A as templates, respectively.

Supplementary Figure 3 Phylogenetic tree of PDCP-1. The phylogenetic tree was constructed using Neighbor-joining method. Homologous protein were retrieved from NCBI nr database with high score using BLAST. The BLAST information of selected sequences are shown in Supplementary Table 1.

Supplementary Figure 4 Domain organization of representative PDZ domain containing proteins of mollusk. Four groups (I ∼ IV) can be divided according to the sequence length and the domain composition. I, the proteins with medium sequence and PDZ/ZM domain; II, the proteins with PDZ/ZM/LIM domain; III, the proteins with short sequence and PDZ/ZM domain; IV, the proteins with long sequence and PDZ/ZM domain.

Supplementary Table 1 BLAST searching results of PDCP-1 in NCBI nr database.

Supplementary Table 2 Amino acid composition (mole percent) of PDCP-1.
