# Supplementary figures and images for: Molecular Characterization of a Novel Shell Matrix Protein With PDZ Domain From Mytilus coruscus
Source: Front Physiol. 2020 Oct 2;11:543758. doi: 10.3389/fphys.2020.543758 (PMC7573561; doi:10.3389/fphys.2020.543758)

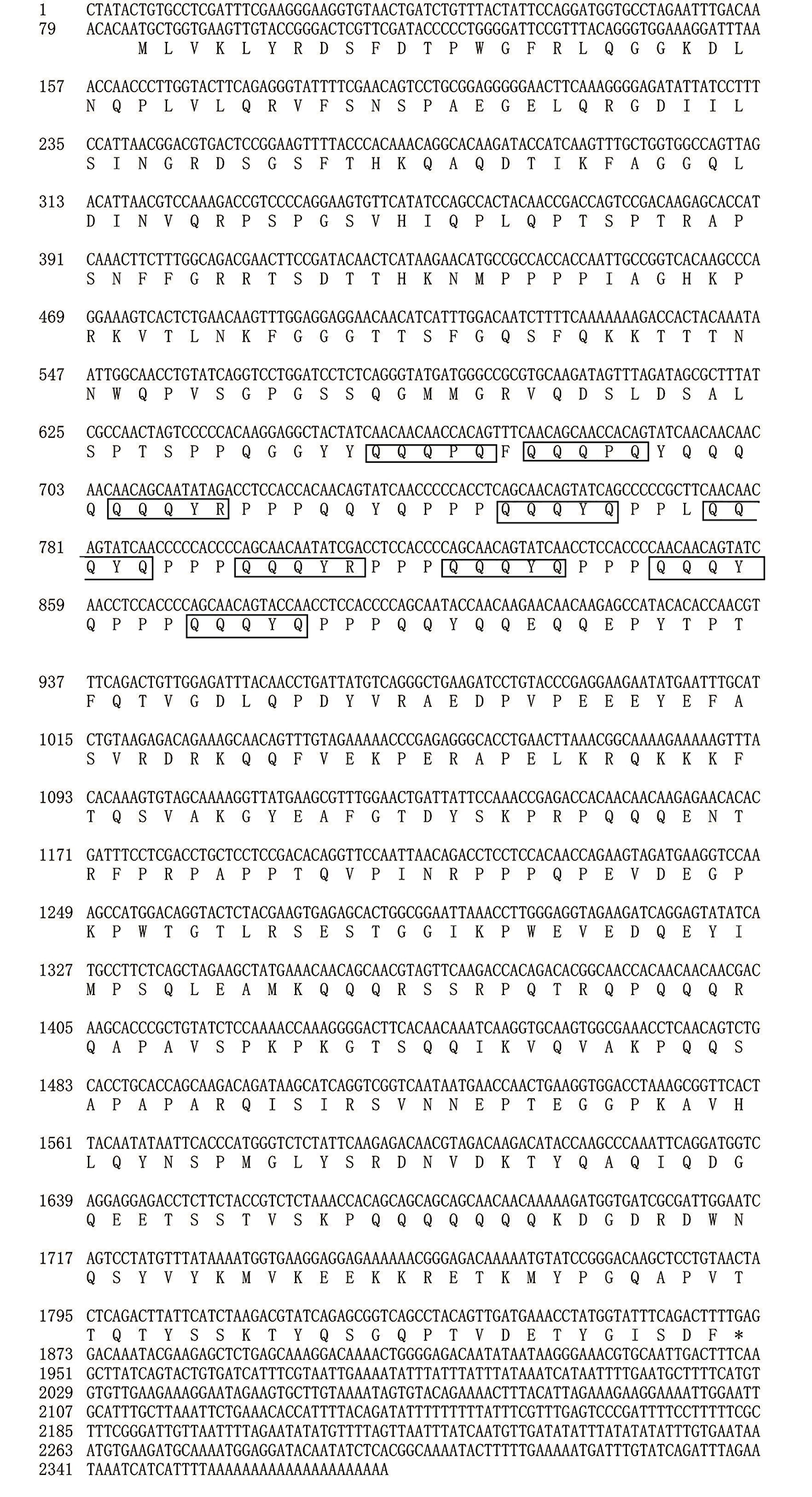

Supplement: Supplementary Figure 1 — Alignment of the cDNA with the deduced amino acid sequence of PDCP-1 (GenBank AKS48142.1). The repetitive “-QQQP(Y)Q(R)” was denoted by frames. *Represents the termination codon. [file Image_1.TIF]

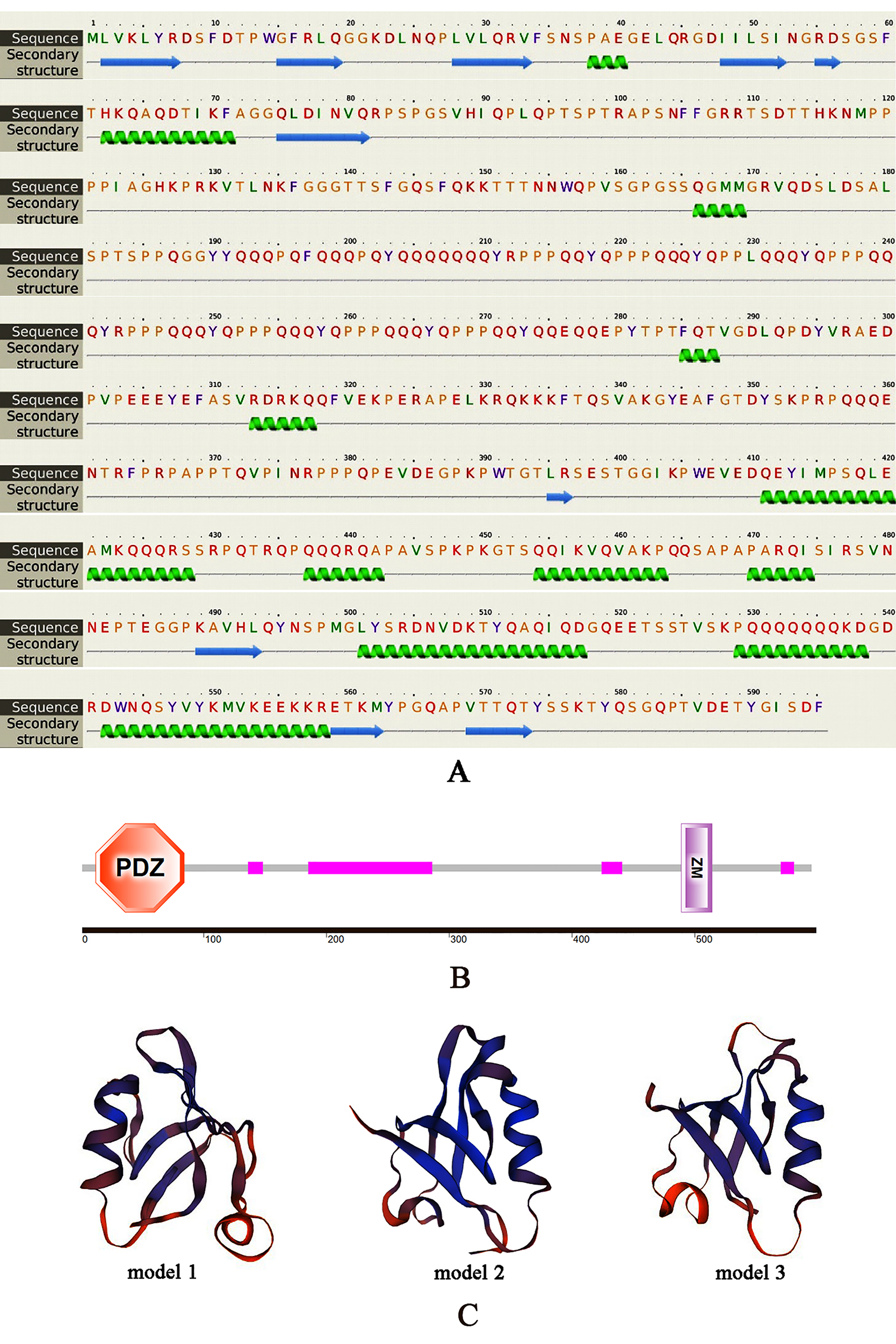

Supplement: Supplementary Figure 2 — Structural features of PDCP-1. (A) The secondary structure of PDCP-1 predicted by Phyre. The regions adopting putative α-helix and β-sheet are represented as spiral and arrow, respectively. (B) Domains of PDCP-1 were predicted by SMART and one PDZ domain and on ZM domain were showed. C: the spatial structure of PDCP-1 predicted by SWISS-MODEL. Model 1∼3 represent the predicted structures with highest score using 1v5l.1.A, 2uzc.5.A and 2q3g.1.A as templates, respectively. [file Image_2.TIF]

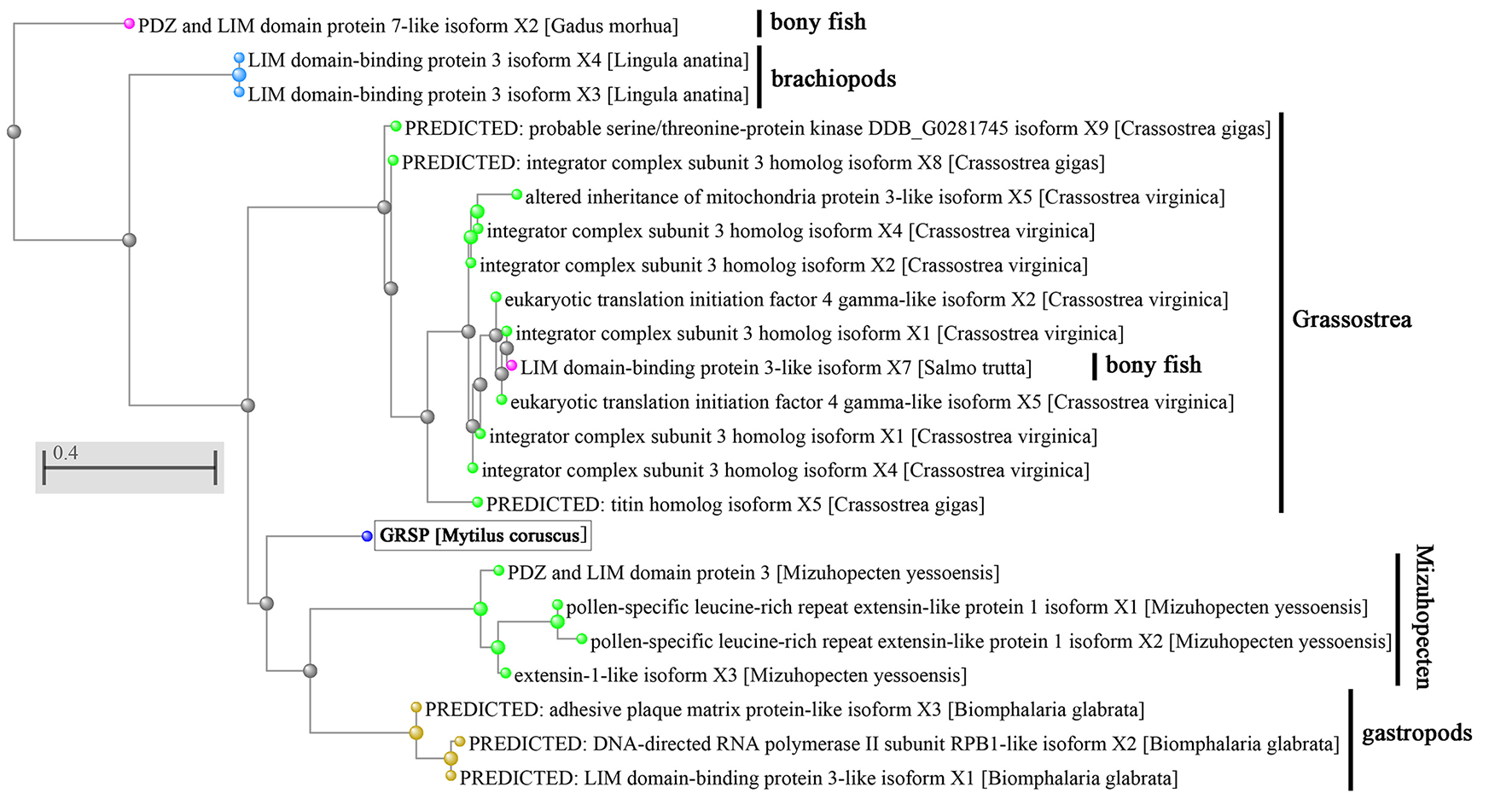

Supplement: Supplementary Figure 3 — Phylogenetic tree of PDCP-1. The phylogenetic tree was constructed using Neighbor-joining method. Homologous protein were retrieved from NCBI nr database with high score using BLAST. The BLAST information of selected sequences are shown in Supplementary Table 1. [file Image_3.TIF]

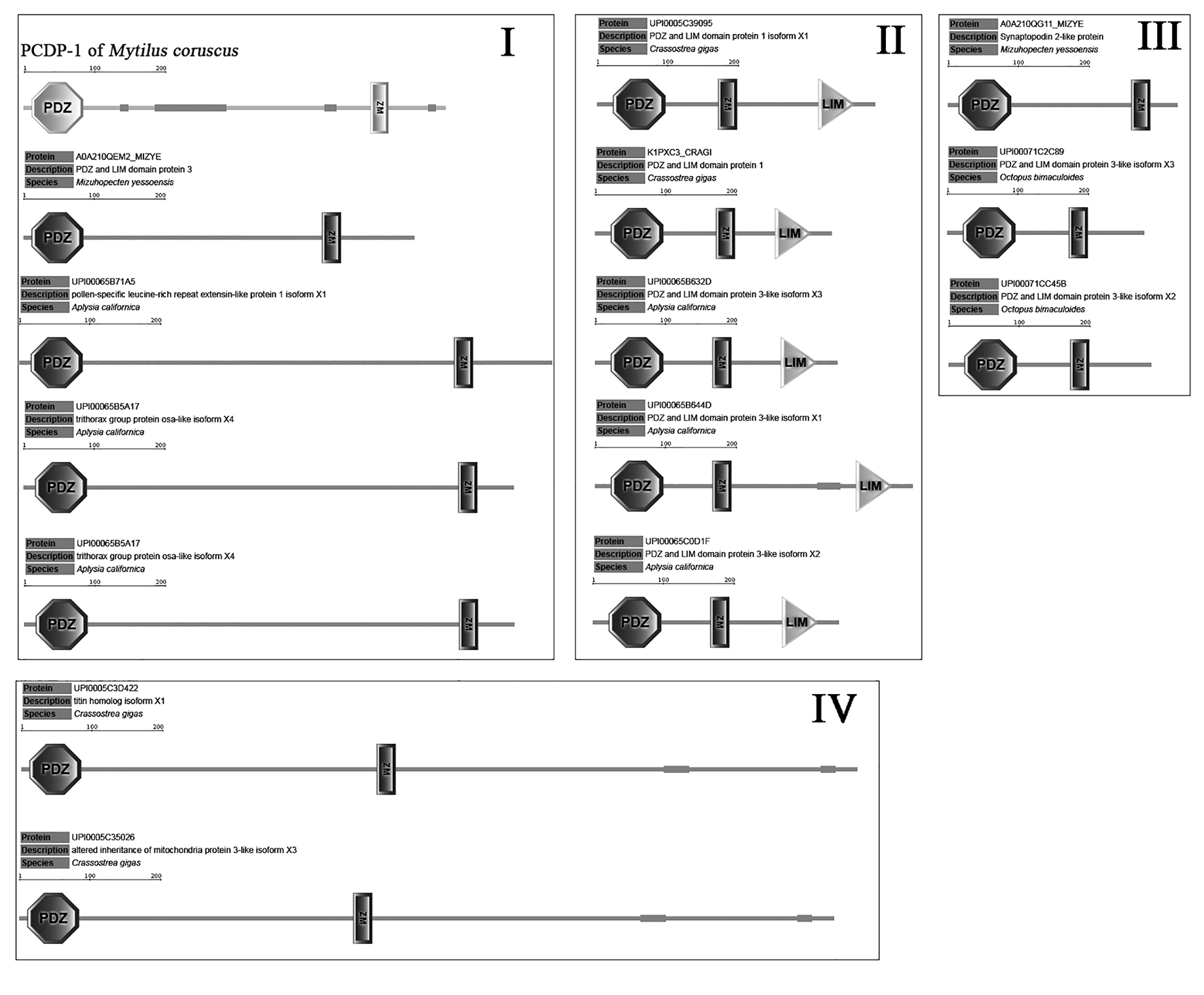

Supplement: Supplementary Figure 4 — Domain organization of representative PDZ domain containing proteins of mollusk. Four groups (I ∼ IV) can be divided according to the sequence length and the domain composition. I, the proteins with medium sequence and PDZ/ZM domain; II, the proteins with PDZ/ZM/LIM domain; III, the proteins with short sequence and PDZ/ZM domain; IV, the proteins with long sequence and PDZ/ZM domain. [file Image_4.TIF]
