# Supplementary material for: Molecular Characterization of a Novel Shell Matrix Protein With PDZ Domain From Mytilus coruscus
Source: Front Physiol. 2020 Oct 2;11:543758. doi: 10.3389/fphys.2020.543758 (PMC7573561; doi:10.3389/fphys.2020.543758)
Supplement: Supplementary Table 1 — BLAST searching results of PDCP-1 in NCBI nr database. [file Table_1.DOCX]

**Supplementary Table 1. BLAST results of GRSP in NCBI nr database**

| **Protein name [species]** | **Max score** | **Total score** | **Query cover** | **E-value** | **Identity** | **Accession** |
| --- | --- | --- | --- | --- | --- | --- |
| PREDICTED: altered inheritance of mitochondria protein 3-like isoform X7 [Crassostrea gigas] | 270 | 270 | 100% | 9.00E-79 | 43.36% | XP_019925971.1 |
| PREDICTED: altered inheritance of mitochondria protein 3-like isoform X6 [Crassostrea gigas] | 260 | 260 | 100% | 9.00E-75 | 40.92% | XP_019925970.1 |
| integrator complex subunit 3 homolog isoform X3 [Crassostrea virginica] | 263 | 263 | 93% | 1.00E-72 | 43.54% | XP_022317991.1 |
| PREDICTED: altered inheritance of mitochondria protein 3-like isoform X3 [Crassostrea gigas] | 263 | 263 | 99% | 1.00E-72 | 42.09% | XP_011438135.1 |
| PREDICTED: titin homolog isoform X1 [Crassostrea gigas] | 251 | 251 | 99% | 3.00E-68 | 40.03% | XP_011438133.1 |
| integrator complex subunit 3 homolog isoform X1 [Crassostrea virginica] | 249 | 249 | 93% | 1.00E-67 | 41.13% | XP_022317989.1 |
| integrator complex subunit 3 homolog isoform X3 [Crassostrea virginica] | 237 | 237 | 80% | 1.00E-63 | 44.76% | XP_022313301.1 |
| PREDICTED: probable serine/threonine-protein kinase DDB_G0281745 isoform X9 [Crassostrea gigas] | 215 | 215 | 100% | 9.00E-59 | 39.74% | XP_019925973.1 |
| integrator complex subunit 3 homolog isoform X1 [Crassostrea virginica] | 223 | 223 | 80% | 1.00E-58 | 41.86% | XP_022313299.1 |
| PREDICTED: titin homolog isoform X2 [Crassostrea gigas] | 223 | 223 | 93% | 1.00E-58 | 37.94% | XP_011438134.1 |
| eukaryotic translation initiation factor 4 gamma-like isoform X2 [Crassostrea virginica] | 221 | 221 | 88% | 8.00E-58 | 38.95% | XP_022317990.1 |
| altered inheritance of mitochondria protein 3-like isoform X5 [Crassostrea virginica] | 213 | 213 | 100% | 9.00E-58 | 38.40% | XP_022313303.1 |
| PREDICTED: integrator complex subunit 3 homolog isoform X8 [Crassostrea gigas] | 205 | 205 | 100% | 9.00E-55 | 37.44% | XP_019925972.1 |
| integrator complex subunit 3 homolog isoform X4 [Crassostrea virginica] | 211 | 211 | 80% | 2.00E-54 | 41.67% | XP_022317992.1 |
| PREDICTED: titin homolog isoform X4 [Crassostrea gigas] | 204 | 204 | 82% | 3.00E-52 | 41.04% | XP_011438136.1 |
| PDZ and LIM domain protein 3 [Mizuhopecten yessoensis] | 195 | 195 | 93% | 2.00E-51 | 38.12% | OWF47196.1 |
| integrator complex subunit 3 homolog isoform X2 [Crassostrea virginica] | 195 | 195 | 74% | 4.00E-49 | 39.31% | XP_022313300.1 |
| PREDICTED: DNA-directed RNA polymerase II subunit RPB1-like isoform X6 [Biomphalaria glabrata] | 168 | 168 | 100% | 1.00E-41 | 34.70% | XP_013067238.1 |
| pollen-specific leucine-rich repeat extensin-like protein 1 isoform X1 [Mizuhopecten yessoensis] | 156 | 156 | 93% | 3.00E-37 | 35.17% | XP_021359996.1 |
| PREDICTED: titin homolog isoform X5 [Crassostrea gigas] | 158 | 158 | 79% | 9.00E-37 | 33.77% | XP_011438137.1 |
| eukaryotic translation initiation factor 4 gamma-like isoform X5 [Crassostrea virginica] | 154 | 154 | 73% | 1.00E-35 | 35.83% | XP_022317994.1 |
| extensin-1-like isoform X3 [Mizuhopecten yessoensis] | 140 | 140 | 93% | 5.00E-32 | 34.88% | XP_021360001.1 |
| PREDICTED: protein lap4-like isoform X3 [Hyalella azteca] | 134 | 340 | 50% | 9.00E-29 | 28.03% | XP_018020967.1 |
| PREDICTED: protein lap4-like isoform X1 [Hyalella azteca] | 133 | 338 | 50% | 1.00E-28 | 28.03% | XP_018020965.1 |
| PREDICTED: protein lap4-like isoform X2 [Hyalella azteca] | 133 | 207 | 50% | 2.00E-28 | 28.03% | XP_018020966.1 |
| pollen-specific leucine-rich repeat extensin-like protein 1 isoform X2 [Mizuhopecten yessoensis] | 129 | 129 | 89% | 5.00E-28 | 32.14% | XP_021360000.1 |
| integrator complex subunit 3 homolog isoform X4 [Crassostrea virginica] | 130 | 130 | 60% | 2.00E-27 | 35.57% | XP_022313302.1 |
| PREDICTED: DNA-directed RNA polymerase II subunit RPB1-like isoform X5 [Biomphalaria glabrata] | 107 | 107 | 81% | 1.00E-20 | 28.24% | XP_013067231.1 |
| afadin [Platynereis dumerilii] | 107 | 107 | 83% | 4.00E-20 | 22.24% | AUG84443.1 |
| protein lap4-like [Limulus polyphemus] | 106 | 362 | 82% | 8.00E-20 | 22.85% | XP_022256926.1 |
| Chain A, LIN-7 homolog B [Homo sapiens] | 95.1 | 95.1 | 14% | 9.00E-20 | 32.18% | 2DKR_A |
| membrane-associated guanylate kinase, WW and PDZ domain-containing protein 2 isoform X7 [Esox lucius] | 105 | 401 | 50% | 1.00E-19 | 25.39% | XP_019900321.1 |
| membrane-associated guanylate kinase, WW and PDZ domain-containing protein 2 isoform X10 [Esox lucius] | 104 | 181 | 49% | 2.00E-19 | 25.16% | XP_012987376.1 |
| membrane-associated guanylate kinase, WW and PDZ domain-containing protein 2 isoform X8 [Esox lucius] | 104 | 398 | 50% | 2.00E-19 | 25.39% | XP_019900322.1 |
| membrane-associated guanylate kinase, WW and PDZ domain-containing protein 2 isoform X8 [Oncorhynchus kisutch] | 104 | 181 | 46% | 3.00E-19 | 22.18% | XP_031687417.1 |
| membrane-associated guanylate kinase, WW and PDZ domain-containing protein 2 isoform X6 [Esox lucius] | 104 | 181 | 50% | 3.00E-19 | 25.39% | XP_019900320.1 |
| membrane-associated guanylate kinase, WW and PDZ domain-containing protein 2 isoform X7 [Oncorhynchus kisutch] | 103 | 408 | 46% | 5.00E-19 | 22.18% | XP_031687416.1 |
| PREDICTED: LIM domain-binding protein 3-like isoform X1 [Biomphalaria glabrata] | 102 | 182 | 98% | 5.00E-19 | 28.24% | XP_013067196.1 |
| membrane-associated guanylate kinase, WW and PDZ domain-containing protein 2 isoform X5 [Esox lucius] | 103 | 178 | 49% | 7.00E-19 | 25.16% | XP_012987372.1 |
| membrane-associated guanylate kinase, WW and PDZ domain-containing protein 2 isoform X3 [Esox lucius] | 103 | 178 | 49% | 7.00E-19 | 25.16% | XP_019900319.1 |
| membrane-associated guanylate kinase, WW and PDZ domain-containing protein 2-like isoform X2 [Parambassis ranga] | 103 | 181 | 57% | 8.00E-19 | 23.20% | XP_028263202.1 |
| membrane-associated guanylate kinase, WW and PDZ domain-containing protein 2 isoform X4 [Esox lucius] | 103 | 400 | 49% | 8.00E-19 | 25.16% | XP_012987371.1 |
| membrane-associated guanylate kinase, WW and PDZ domain-containing protein 2-like isoform X1 [Parambassis ranga] | 103 | 380 | 57% | 9.00E-19 | 23.20% | XP_028263201.1 |
| LOW QUALITY PROTEIN: membrane-associated guanylate kinase, WW and PDZ domain-containing protein 2 [Oreochromis niloticus] | 103 | 181 | 43% | 9.00E-19 | 25.64% | XP_025765253.1 |
| membrane-associated guanylate kinase, WW and PDZ domain-containing protein 2-like isoform X5 [Parambassis ranga] | 102 | 180 | 57% | 9.00E-19 | 23.20% | XP_028263206.1 |
| membrane-associated guanylate kinase, WW and PDZ domain-containing protein 2 isoform X1 [Esox lucius] | 102 | 393 | 49% | 1.00E-18 | 25.16% | XP_019900317.1 |
| membrane-associated guanylate kinase, WW and PDZ domain-containing protein 2-like isoform X3 [Parambassis ranga] | 102 | 379 | 53% | 1.00E-18 | 25.00% | XP_028263203.1 |
| membrane-associated guanylate kinase, WW and PDZ domain-containing protein 2 isoform X2 [Esox lucius] | 102 | 393 | 49% | 1.00E-18 | 25.16% | XP_019900318.1 |
| PREDICTED: membrane-associated guanylate kinase, WW and PDZ domain-containing protein 2-like [Cyprinodon variegatus] | 102 | 180 | 46% | 1.00E-18 | 24.30% | XP_015247757.1 |
| membrane-associated guanylate kinase, WW and PDZ domain-containing protein 2-like [Oreochromis aureus] | 102 | 180 | 43% | 1.00E-18 | 25.64% | XP_031593190.1 |
| membrane-associated guanylate kinase, WW and PDZ domain-containing protein 2 isoform X5 [Sparus aurata] | 102 | 424 | 53% | 1.00E-18 | 23.00% | XP_030296247.1 |
| membrane-associated guanylate kinase, WW and PDZ domain-containing protein 2-like isoform X6 [Parambassis ranga] | 102 | 179 | 43% | 1.00E-18 | 25.00% | XP_028263207.1 |
| membrane-associated guanylate kinase, WW and PDZ domain-containing protein 2-like isoform X7 [Parambassis ranga] | 102 | 179 | 43% | 2.00E-18 | 25.00% | XP_028263208.1 |
| membrane-associated guanylate kinase, WW and PDZ domain-containing protein 2-like isoform X7 [Oncorhynchus mykiss] | 102 | 357 | 49% | 2.00E-18 | 23.47% | XP_021460869.1 |
| membrane-associated guanylate kinase, WW and PDZ domain-containing protein 2-like isoform X2 [Oncorhynchus mykiss] | 102 | 350 | 49% | 2.00E-18 | 23.47% | XP_021460837.1 |
| membrane-associated guanylate kinase, WW and PDZ domain-containing protein 2-like isoform X3 [Oncorhynchus mykiss] | 102 | 357 | 49% | 2.00E-18 | 23.47% | XP_021460841.1 |
| PDZ and LIM domain protein 7-like isoform X2 [Gadus morhua] | 100 | 147 | 70% | 3.00E-18 | 31.65% | XP_030234569.1 |
| PDZ and LIM domain protein 7-like isoform X1 [Gadus morhua] | 100 | 100 | 45% | 3.00E-18 | 31.65% | XP_030234568.1 |
| LIM domain-binding protein 3-like isoform X10 [Salmo trutta] | 100 | 100 | 41% | 4.00E-18 | 31.30% | XP_029554073.1 |
