# Supplementary material for: Molecular Characterization of a Novel Shell Matrix Protein With PDZ Domain From Mytilus coruscus
Source: Front Physiol. 2020 Oct 2;11:543758. doi: 10.3389/fphys.2020.543758 (PMC7573561; doi:10.3389/fphys.2020.543758)
Supplement: Supplementary Table 2 — Amino acid composition (mole percent) of PDCP-1. [file Table_2.DOCX]

**Supplementary Table 2: Amino acid composition (mole percent) of GRSP**

| Amino acid |  | Mole percent |
| --- | --- | --- |
| Gln (Q) |  | 18.2% |
| Pro (P) |  | 13.6% |
| Ser (S) |  | 7.2% |
| Gly (G) |  | 6.4% |
| Thr (T) |  | 6.4% |
| Lys (K) |  | 5.9% |
| Arg (R) |  | 5.4% |
| Glu (E) |  | 4.9% |
| Tyr (Y) |  | 4.7% |
| Val (V) |  | 4.7% |
| Asp (D) |  | 4.2% |
| Ala (A) |  | 3.7% |
| Leu (L) |  | 3.4% |
| Phe (F) |  | 3.0% |
| Asn (N) |  | 2.7% |
| Ile (I) |  | 2.5% |
| Met (M) |  | 1.5% |
| His (H) |  | 0.8% |
| Trp (W) |  | 0.8% |
| Cys (C) |  | 0.0% |
| Pyl (O) |  | 0.0% |
| Sec (U) |  | 0.0% |
